# Unmodified mRNA in LNPs constitutes a competitive technology for prophylactic vaccines

**DOI:** 10.1038/s41541-017-0032-6

**Published:** 2017-10-19

**Authors:** Johannes Lutz, Sandra Lazzaro, Mohamed Habbeddine, Kim Ellen Schmidt, Patrick Baumhof, Barbara L. Mui, Ying K. Tam, Thomas D. Madden, Michael J. Hope, Regina Heidenreich, Mariola Fotin-Mleczek

**Affiliations:** 1 CureVac AG, Paul-Ehrlich-Str. 15, 72076 Tübingen, Germany; 2 Acuitas Therapeutics, Vancouver, BC V6T 1Z3 Canada

## Abstract

mRNA represents a promising new vaccine technology platform with high flexibility in regard to development and production. Here, we demonstrate that vaccines based on sequence optimized, chemically unmodified mRNA formulated in optimized lipid nanoparticles (LNPs) are highly immunogenic and well tolerated in non-human primates (NHPs). Single intramuscular vaccination of NHPs with LNP-formulated mRNAs encoding rabies or influenza antigens induced protective antibody titers, which could be boosted and remained stable during an observation period of up to 1 year. First mechanistic insights into the mode of action of the LNP-formulated mRNA vaccines demonstrated a strong activation of the innate immune response at the injection site and in the draining lymph nodes (dLNs). Activation of the innate immune system was reflected by a transient induction of pro-inflammatory cytokines and chemokines and activation of the majority of immune cells in the dLNs. Notably, our data demonstrate that mRNA vaccines can compete with licensed vaccines based on inactivated virus or are even superior in respect of functional antibody and T cell responses. Importantly, we show that the developed LNP-formulated mRNA vaccines can be used as a vaccination platform allowing multiple, sequential vaccinations against different pathogens. These results provide strong evidence that the mRNA technology is a valid approach for the development of effective prophylactic vaccines to prevent infectious diseases.

## Vaccine technology: Repurposing the genetic messenger

Vaccines based on mRNA provoke strong immune responses after a single dose. mRNA is commonly known as the ‘genetic messenger’ cousin of DNA and a crucial mediator of protein production. Now, research led by Mariola Fotin-Mleczek, of Germany’s CureVac AG, demonstrates that mRNA can be developed to produce virus fragments, called antigens, that can prime a vaccinee’s immune system against a pathogen. Testing their vaccine platform, the team created mRNA coding for rabies and influenza antigens, and used intramuscular injection to inoculate non-human primates. A single dose elicited strong immune responses, which the team then successfully maintained through booster vaccinations for an observation period of 1 year. The responses outperformed those of licensed vaccines against rabies and influenza type H3N2. This research shows that mRNA has promise as a versatile, cost-effective, rapidly scalable vaccine technology.

## Introduction

The introduction of prophylactic vaccination has been one of the most effective medical interventions to fight and eradicate infectious diseases. Despite its great successes, the continuous threat of infectious agents for which no vaccine exists and the introduction of new pathogens into the human population emphasize the need for the development of novel safe vaccines and even vaccine platforms capable of rapidly responding to those needs. Ideally, these vaccine platforms should be highly versatile at minimal development and production costs.

Vaccines based on mRNA could meet these requirements because they offer multifaceted advantages including flexible antigen design, a cost-effective manufacturing process allowing for parallel production of multiple mRNA vaccines^1–5^ and rapid manufacturing, which could be of crucial importance during pandemics.^6^

Using exclusively unmodified nucleosides, we have demonstrated that non-replicating mRNA vaccines are immunogenic and capable of inducing protection against lethal rabies and influenza virus infections after intradermal vaccination (i.d.) in mice, rats, ferrets, and pigs.^7,8^ These vaccines contained free and protamine-complexed mRNA to support both strong antigen expression and immunostimulation.^9^ This approach was specifically optimized for i.d. administration and showed lower efficacy when given by the intramuscular route (i.m.) as preferred route for prophylactic vaccination, highlighting the important role of formulation in vaccine design. Studies with self-amplifying mRNA vaccines investigated the effect of different formulations such as lipid-nanoparticles (LNP)^10^ or cationic nanoemulsion (CNE)^11^ on the immunogenicity of mRNA vaccines. Self-amplifying vaccines benefited from formulation with LNP or CNE and were able to induce protective antibody titers. In contrast, non-replicating mRNA did not induce any detectable antibody titers even when formulated with CNE. These data reveal a gap between self-amplifying and non-replicating mRNA, which cannot be closed only by formulation. This demonstrates a clear need to optimize the mRNA itself to obtain sufficient expression levels. To this end, different approaches of optimization are pursued.

In recent publications chemically modified nucleosides were used to reach sufficient antigen expression,^12–15^ which is in contrast to our proprietary mRNA technology, which employs sequence optimization and selected untranslated regions (UTRs) to achieve high antigen expression.^16^ In these studies, mice were vaccinated i.m. with LNP-formulated mRNA vaccines inducing protection against Zika virus challenge infections,^12^ and protection against placental damage and fetal demise in challenged pregnant mice.^13^ Importantly, protective efficacy against Zika virus challenge infections was also demonstrated in non-human primates (NHPs) after i.d. vaccination.^14^ Immunogenicity data in NHPs are absolutely desirable taking into account the experiences with early DNA vaccines, for which efficacy could not be translated from mice to NHPs. The study by Pardi et al. demonstrates that non-replicating mRNA is able to induce antibody titers in NHPs. To this end, they applied ten separate i.d. injections distributed on the back of the animals. For prophylactic vaccination single injections are highly desirable with i.m. application being the routinely used route. Notably, previous studies have demonstrated lower vaccine immunogenicity after i.m. compared to i.d. vaccination, which could be due to the lower frequency of antigen-presenting cells in the muscle compared to the skin. Meanwhile, induction of protective immune responses against two different influenza strains could be demonstrated also after i.m. administration in NHPs and humans, again using LNP-formulated mRNA vaccines based on chemically modified nucleosides.^15^ In summary, while those results definitely established mRNA as an appropriate tool for vaccination in research, further questions have to be addressed to finally prove it to be a competitive platform for vaccine development.

In the present study, we compare mRNA vaccines to licensed vaccines based on inactivated virus and demonstrate that unmodified mRNA, formulated in optimized LNPs, fulfills all the key requirements to be a viable vaccine platform for human prophylaxis. We show for the first time that a single intramuscular injection of non-replicating mRNA vaccines induces functional antibody titers in NHPs comparable to or even higher than vaccination with a full human dose of licensed vaccines based on inactivated virus. The immune responses could be boosted and stayed stable during an observation period of up to 1 year. Moreover, we demonstrated the potential of mRNA vaccines as a platform approach by successfully vaccinating the same animals with multiple vaccines based on the same technology.

## Results

### Unmodified mRNA in LNPs supports high antigen expression in muscle cells and induces a pro-inflammatory environment

The induction of strong immune responses after intramuscular injection of mRNA represents a very high hurdle due to a low infiltration of muscle tissue by immune cells and the lack of costimulatory molecules and optimal antigen presentation on muscle cells. Thus, potent intramuscular mRNA vaccines must allow high antigen expression and presentation and induce strong immunostimulatory signals to improve immune cell infiltration. In the last years LNPs have been extensively explored for in vivo delivery of mRNA^17^ and have shown to be a promising non-viral delivery system. Therefore, we used in our study unmodified, sequence-optimized mRNA formulated with an optimized LNP consisting of an ionizable amino lipid, phospholipid, cholesterol and a polyethylene glycol (PEG) containing lipid.

**Fig. 1:**
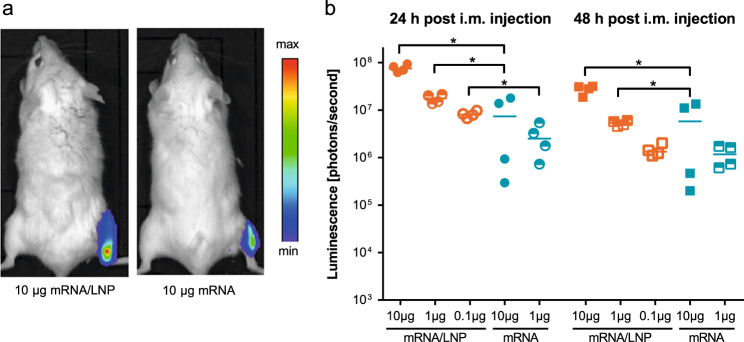
LNP formulation enhances protein expression in vivo after i.m. application. BALB/c mice (*n = *4/group) were injected i.m. with non-formulated (mRNA) or LNP-formulated (mRNA/LNP) PpLuc mRNA. **a** 24 and 48 h after injection, luciferase expression was visualized in vivo by optical imaging. **b** Quantification of luciferase expression by luminescence. Values from individual mice (dots) and the median (solid lines) are reported for each group. Statistical analysis was performed using Mann–Whitney test. *p = 0.0286

We first analyzed the expression of the LNP-formulated mRNA (mRNA/LNP) in muscle tissue using mRNA encoding the reporter *Photinus pyralis* luciferase (PpLuc). Mice injected with PpLuc mRNA/LNP showed at 24 and 48 h significantly higher intramuscular luciferase expression than animals that had received non-formulated PpLuc mRNA (Fig. 1).

**Fig. 2:**
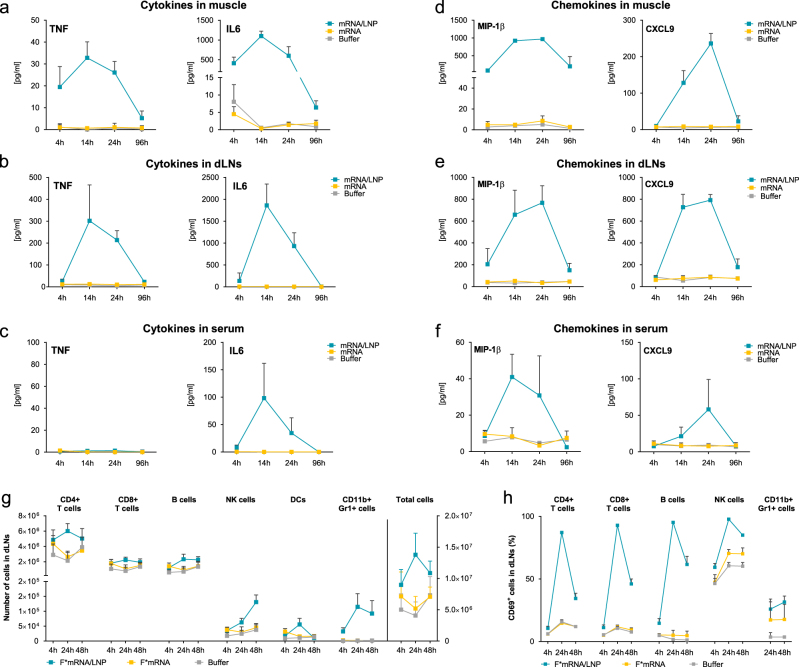
LNP-formulated mRNA induces a pro-inflammatory environment. **a**–**f** BALB/c mice (*n = *6/group) were vaccinated i.m. with 10 µg non-formulated (mRNA) or LNP-formulated RABV-G mRNA (mRNA/LNP), or with buffer. Muscle tissues, dLNs and serum samples were isolated and cytokine (**a**–**c**) or chemokine content (**d**–**f**) was measured in protein lysates and sera by cytometric bead array (CBA). **g**, **h** BALB/c mice (*n = *3/group) were injected i.m. in both legs with 10 µg non-formulated (F*mRNA) or LNP-formulated F*mRNA (F*mRNA/LNP), or with buffer. Right and left dLNs were isolated and analyzed separately by flow cytometry. Numbers of each cell population (**g**) and frequency of activated immune cells (**h**) in the dLNs are shown. Values represent mean of six samples with SD

To gain first mechanistic insights into the mode of action of mRNA/LNP vaccines we evaluated the activation of the innate immune system, which is required to mount efficacious adaptive immune responses after vaccination.^18^ To this end, cytokine and chemokine concentrations were analyzed at the injection site and in the draining lymph nodes (dLNs) after i.m. administration of LNP-formulated mRNA encoding the rabies virus glycoprotein G (RABV-G). In contrast to buffer or non-formulated mRNA, LNP-formulated mRNA induced a pronounced but transient release of the pro-inflammatory cytokines tumor necrosis factor (TNF) and IL-6 (Fig. 2a, b) with peak concentrations at 14 h after injection. Importantly, the increase in cytokine concentrations was predominantly local with systemic TNF concentrations below detection limit and only a transient increase in IL-6 concentrations. The IL-6 concentrations remained 10-fold lower in the serum compared to the injection site and returned to baseline at 96 h after injection (Fig. 2c). The higher concentrations at the injection site and in the dLNs strongly suggest a local production of these cytokines. However, as LNPs are efficient delivery vehicles to the liver,^19^ a contribution of the liver to the increased systemic IL-6 concentrations cannot be excluded. Pro-inflammatory chemokines, which are known to contribute to the recruitment and subsequent activation of distinct immune cells, were also transiently induced at the injection site and in the dLNs by LNP-formulated mRNA (Fig. 2d, e). Among the strongly upregulated chemokines were MIP-1β, which plays a pivotal role in the chemotaxis of macrophages, monocytes and NK cells, and CXCL-9, which recruits T cells, NK cells and NKT cells to the site of inflammation.^20^ Moreover, there was a transient elevation in the concentrations of MCP-1, MIP-1α, and CXCL1, which attract a variety of immune cells such as monocytes, macrophages, dendritic cells and neutrophils (data not shown). We also observed a transient increase in serum concentrations of the chemokines described above, but to a much lower extent compared to those detected at the injection site or in the dLNs (Fig. 2f).

To elucidate whether the pro-inflammatory environment translates into activation and changes in the composition of immune cells, we next analyzed the number and activation status of leukocytes in the dLNs. To ensure that any observed effect was independent of the mRNA-encoded protein, we used a fluorescently labeled mRNA that cannot be translated (F*mRNA). Intramuscular injection of the LNP-formulated F*mRNA induced a strong increase in cellularity, which was absent after injection of unformulated F*mRNA (Fig. 2g). The strongest elevation in cell numbers was observed 24 h after injection, except for NK cells which increased over time. CD11b^+^ Gr1^+^ cells, consisting mainly of monocytes and granulocytes, accounted for the largest increase in leukocytes. Within 4 h after injection of LNP-formulated mRNA, we observed a 10-fold increase in CD11b^+^ Gr1^+^ cells compared to mice treated with non-formulated mRNA. The increase in cellularity in dLNs was accompanied by a strong activation of both adaptive and innate immune cells, which peaked at 24 h after injection, when more than 90% of the T and B cells expressed the activation marker CD69 (Fig. 2h). Taken together, these results suggest that i.m. injection of LNP-formulated mRNA vaccines induces a broad but transient local immunostimulatory milieu, which is relevant for the induction of strong adaptive immune responses.

### LNP-formulated mRNA induces strong humoral and cellular responses in mice

**Fig. 3:**
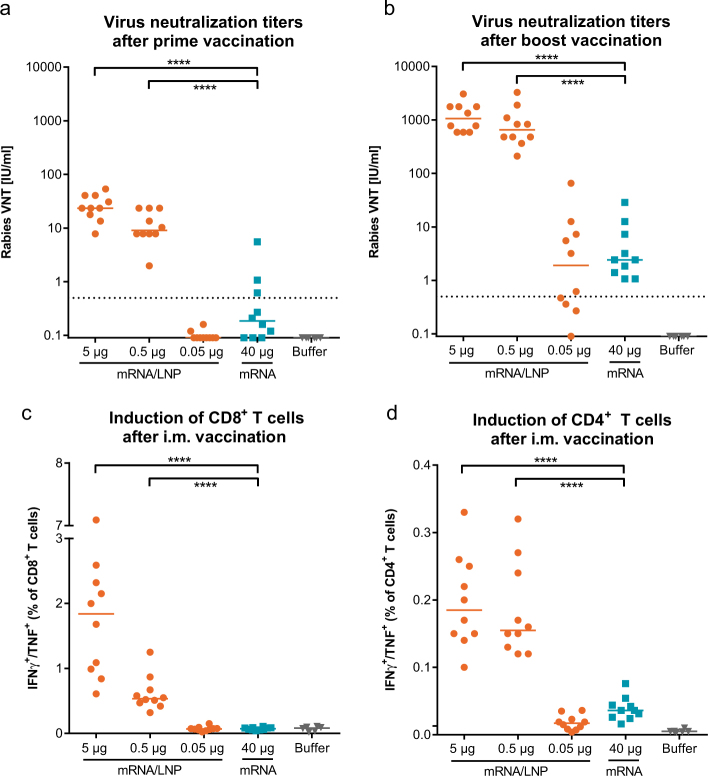
LNP-formulated mRNA vaccine induces humoral and cellular immune responses in mice. BALB/c mice (*n = *10/group) were vaccinated i.m. at days 0 and 21 with non-formulated RABV-G mRNA (mRNA), LNP-formulated RABV-G mRNA (mRNA/LNP) or with buffer. Rabies VNTs were analyzed in the sera 3 weeks after prime (**a**) and 2 weeks after boost vaccination (**b**). Splenocytes were stimulated 2 weeks after boost vaccination with an overlapping peptide library covering the RABV-G protein. Frequencies of antigen-specific, multifunctional (IFN-γ^+^/TNF^+^) CD8^+^ (**c**) and CD4^+^ (**d**) T cells were detected by intracellular cytokine staining. Values from individual mice (dots) and the median (solid lines) are reported for each group. The dashed line indicates the conventionally defined protective titers of 0.5 IU/ml for rabies VNTs. Statistical analysis was performed using Mann–Whitney test. ****p < 0.0001

Having demonstrated that LNP-formulated, optimized mRNA administered i.m. supports high antigen expression and activates innate immunity, we vaccinated BALB/c mice with the RABV-G mRNA that has previously demonstrated protective efficacy after i.d. application.^7^ A prime vaccination with a dose of 0.5 µg LNP-formulated RABV-G mRNA already induced virus neutralization titers (VNTs) above the WHO reference titer of 0.5 IU/ml, which is used as correlate of protection in humans^21^ (Fig. 3a). These neutralizing antibody titers increased more than 50-fold after a second vaccination (Fig. 3b). The VNTs induced by 0.5 µg of LNP-formulated mRNA were more than 250-fold higher (median 650 IU) compared to titers induced by 40 µg of non-formulated mRNA (median 2.4 IU). Additionally, LNP-formulated mRNA led to stronger cellular immune responses, which was reflected by significantly higher frequencies of antigen-specific multifunctional CD4^+^ and CD8^+^ T cells (Fig. 3c, d) compared to non-formulated mRNA.

### Intramuscular vaccination with LNP-formulated mRNA leads to long-lived humoral responses in NHPs

**Fig. 4:**
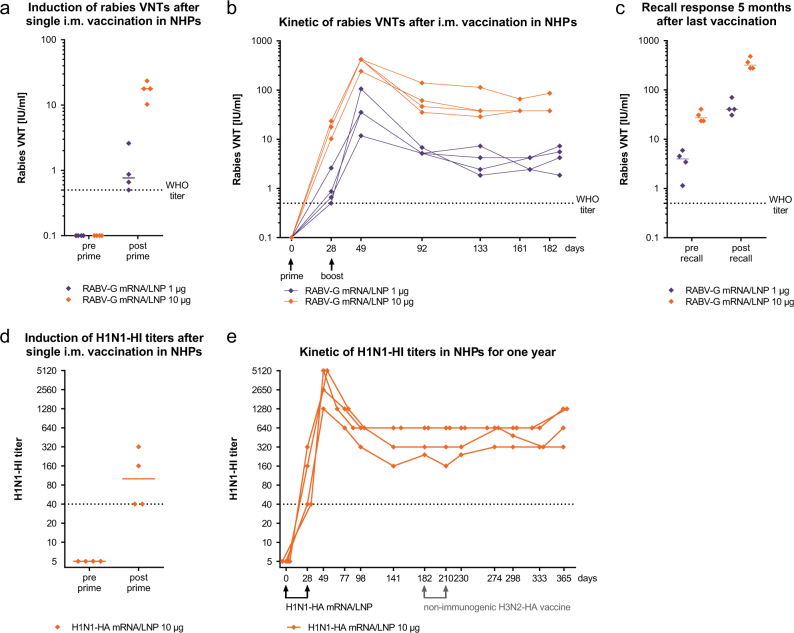
mRNA vaccines induce protective antibody titers against RABV-G and H1N1-HA in NHPs. NHPs (*n* = 2 m, 2 f per group) were vaccinated i.m. with the respective mRNA/LNP vaccines. **a** Rabies VNTs in the sera before and 28 days after prime vaccination with RABV-G mRNA/LNP. **b** Kinetic of rabies VNTs in the sera. **c** Rabies VNTs in the plasma before and 5 days after recall vaccination, which was performed 5 months after boost vaccination. **d** H1N1-HI titers in the sera of NHPs before and 28 days after prime vaccination with H1N1-HA mRNA/LNP. **e** Kinetic of H1N1-HI titers after H1N1-HA mRNA/LNP vaccinations. Animals received also an explorative H3N2-HA mRNA/non-LNP vaccine at days 182 and 210, which turned out to be not immunogenic. Values from individual animals (dots) are reported for each group. The median (solid lines) is reported in **a**, **c**, **d**. Dashed lines indicate the conventionally defined protective titers of 0.5 IU/ml for rabies VNTs and 1:40 for HI

Next, we tested whether the strong immunogenicity of mRNA vaccines in mice translates into higher-order species. To this end, we vaccinated cynomolgus monkeys with the LNP-formulated unmodified RABV-G mRNA. A single i.m. immunization with 1 µg LNP-formulated RABV-G mRNA already induced robust VNTs at or above the WHO titer of 0.5 IU/ml in all animals at day 28 after prime vaccination (Fig. 4a). The observed immunogenicity was dose dependent with a 10-fold higher mRNA dose yielding 10-fold higher VNTs.

To evaluate whether the primary responses could be boosted a second vaccination with the RABV-G mRNA/LNP vaccine was performed at day 28 resulting in a 20-fold increase in VNTs. Monitoring the antibody titers for 6 months we could demonstrate that after an initial decline neutralizing titers stabilized at a level of about 40 IU/ml for the 10 µg mRNA dose and about 4 IU/ml for the 1 µg mRNA dose (Fig. 4b). To demonstrate the existence of B cell memory we vaccinated the same animals 5 months after completed vaccination a third time with RABV-G mRNA/LNPs (recall vaccination) and measured antibody titers 5 days later. In both dose groups we observed a very rapid 10-fold increase in VNTs, demonstrating the induction of a strong recall-response by the mRNA vaccine (Fig. 4c).

To confirm the platform potential of our mRNA vaccination technology, we produced a vaccine encoding hemagglutinin (HA) of the influenza virus strain H1N1pdm09. Naïve NHPs were vaccinated with 10 µg of the H1N1-HA mRNA/LNP vaccine and functional antibodies against the influenza H1N1 virus were measured by a hemagglutination inhibition (HI) assay. After just a single vaccination, we observed H1N1-HI titers at or above 1:40, which is used as correlate of protection in humans^21^ (Fig. 4d). A second dose of the H1N1-HA mRNA/LNP vaccine strongly increased the H1N1-HI titers, which again after an initial decline stabilized at a titer of about 640 (Fig. 4e). Importantly, all vaccinated animals maintained H1N1-HI titers clearly above the protective limit for 5 months starting after prime vaccination pointing to longevity of the mRNA-mediated humoral response. The NHPs were then used to test an explorative non-LNP formulation and were vaccinated at days 182 and 210 with an mRNA encoding HA of the influenza virus strain H3N2 in an explorative non-LNP formulation. In contrast to the LNP formulation, the non-LNP formulation did not induce antigen-specific humoral responses and no H3N2-HI titers could be detected after prime or boost vaccination (Supplementary Fig. 1). The H1N1-HI titers were not changed by the non-immunogenic H3N2-HA vaccination. A continued follow-up of the H1N1-HI titers demonstrated stable H1N1-HI titers induced by the primary H1N1-HA mRNA/LNP vaccination until the end of the observation period of 1 year.

### LNP-formulated mRNA vaccines are capable of competing with licensed vaccines in NHPs

**Fig. 5:**
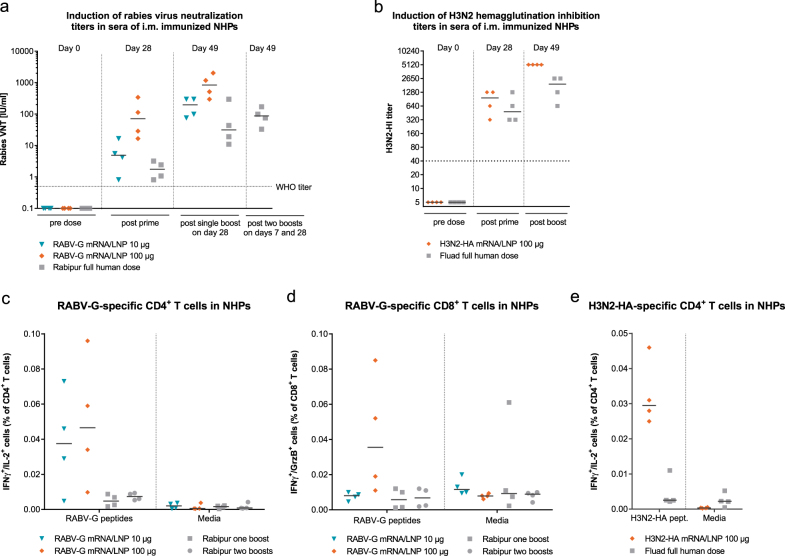
mRNA vaccines induce in NHPs stronger humoral and cellular responses against rabies or H3N2 influenza than licensed vaccines. **a** Rabies VNTs in the sera of NHPs (*n* = 2 m, 2 f per group) vaccinated with RABV-G mRNA/LNP at days 0 and 28, or with the inactivated rabies virus vaccine Rabipur^®^ at days 0 and 28 (one boost) or days 0, 7, and 28 (two boosts). **b** H3N2-HI titers in the sera of NHPs (*n* = 2 m, 2 f per group) vaccinated at days 0 and 28 with H3N2-HA mRNA/LNP or the adjuvanted vaccine Fluad^®^. **c**, **d** Frequencies of RABV-G-specific IFNγ^+^/IL-2^+^ CD4^+^ and IFNγ^+^/GrzB^+^ CD8 T cells in the blood 7 days after the last vaccination (day 35). PBMCs were either stimulated with an overlapping peptide library covering the RABV-G protein (RABV-G peptides) or unstimulated (media) and analyzed by ICS. **e** Frequencies of H3N2-HA-specific IFNγ^+^/IL-2^+^ CD4^+^ cells in the blood 7 days after the last vaccination (day 35). PBMCs were either stimulated with an overlapping peptide library covering the H3N2-HA protein (H3N2-HA peptides) or unstimulated (media) and analyzed by ICS. Gating is shown in Supplementary Fig. 2. Values from individual animals (dots) and the median (solid lines) are reported for each group. Dashed lines indicate the conventionally defined protective titers of 0.5 IU/ml for rabies VNTs and 1:40 for HI

To test whether mRNA vaccines represent a competitive platform for vaccine development, we compared in NHPs the immunogenicity of the RABV-G mRNA/LNP vaccine and an H3N2-HA mRNA/LNP vaccine to already licensed vaccines based on inactivated virus, namely Rabipur® and Fluad®. We compared the potency of the vaccines to prime effective immune responses, as well as their capability to boost immune responses. The RABV-G mRNA/LNP vaccine induced after a single vaccination neutralizing antibody titers above 0.5 IU/ml, which were comparable (for the 10 µg mRNA dose) or higher (for the 100 µg mRNA dose) than the VNTs induced by a full human dose of Rabipur® (Fig. 5a). Four weeks after a single shot vaccination median VNTs measured for mRNA vaccines were 4.9 IU/ml for the 10 µg dose and 71.2 IU/ml for the 100 µg dose, compared to 1.8 IU/ml for Rabipur®. Antibody titers of all groups benefited from a second vaccination given at day 28, and VNTs (measured on day 49) induced by 100 µg of mRNA vaccine reached a median of 842 IU/ml, outperforming Rabipur® more than 20-fold (median 31.3 IU/ml). As the recommended pre-exposure vaccination schedule for Rabipur® implies three administrations conducted on days 0, 7 and 28,^22^ we also included this group. However, the additional vaccination with Rabipur® on day 7 did also not result in higher VNTs at day 49 than after two mRNA vaccine administrations. These data suggest that the vaccination schedule with two injections of the LNP-formulated mRNA vaccine is sufficient to induce protection against rabies infections. For the 100 µg dose of LNP-formulated RABV-G mRNA, which exceeded the WHO titer of 0.5 IU/ml by 20 to 200-fold at day 28, even a single administration may already be sufficient to induce protective and sustained antibody titers.

The mRNA technology was also competitive when we compared an H3N2-HA mRNA/LNP vaccine in NHPs to the potent flu vaccine Fluad® for the season 2016/17. Fluad® contains the surface antigens HA and neuraminidase of the influenza strains H1N1, H3N2 and B/Brisbane, as well as the adjuvant MF59C.1. A single dose of the H3N2-HA mRNA/LNP vaccine was sufficient to induce H3N2-HI titers, which were above the titer of 1:40 and comparable to titers induced by a full human dose of Fluad®. A second dose of both vaccines further increased the titers, to yet higher titers for the mRNA/LNP vaccine.

Finally, we compared the mRNA/LNP vaccines with the licensed vaccines in their capability to induce T cell responses in NHPs. An intracellular cytokine staining (ICS) analysis of PBMCs of the rabies-vaccinated animals at day 35, i.e., seven days after the last vaccination, detected specific cellular responses after mRNA/LNP vaccination but not in Rabipur®-vaccinated animals. RABV-G-specific multifunctional IFN-γ^+^/IL-2^+^ CD4^+^ T cells, which are associated with strong anti-viral responses,^23^ were observed for both mRNA vaccine doses, whereas RABV-G-specific IFN-γ^+^/GrzB^+^ CD8^+^ T cells, which are dedicated cytolytic cells,^24^ were detected only in animals receiving the 100 µg dose (Fig. 5c, d, Supplementary Fig. 2). In the influenza-vaccinated animals H3N2-HA-specific IFN-γ^+^/IL-2^+^ CD4^+^ T cells were observed at day 35 only for the mRNA/LNP vaccine but not for Fluad® (Fig. 5e **)**. H3N2-HA-specific CD8^+^ T cell responses were absent in both cases.

### mRNA vaccines exhibit a favorable safety profile

Favorable tolerability and safety are critical requirements for prophylactic vaccines. To address these aspects we analyzed in the NHPs the reactogenicity at the injection site, systemic cytokine and chemokine concentrations, body weight, and body temperature after i.m. vaccination with 1, 10, and 100 µg LNP-formulated mRNA. Injection sites showed only slight erythema and/or edema in some of the animals, which resolved 1 to 6 days after injection (Table 1). No pain on palpation was observed. Systemic cytokine concentrations after vaccination stayed for most animals and time points below the detection limit (IL-1β, IL-4, IL-5, IFN-γ and TNF) or did not increase significantly above pre-dose concentrations (IL-2, IL-8 and G-CSF) (Supplementary Fig. 3a and data not shown). IL-6 was absent in almost all animals that received the 1 or 10 µg dose. Vaccination with the 100 µg dose induced at 24 h in all animals a low increase in IL-6 concentrations. Body temperatures, which varied considerably among animals and studies, did not display obvious differences between NHPs that had received the mRNA/LNP vaccines or the licensed vaccines (Supplementary Fig. 3b). Similarly, no changes in food consumption or body weight were associated with any dose (data not shown). These data demonstrate a good tolerability of the mRNA/LNP vaccines in NHPs and confirm the observations in mice, where systemic release of TNF or IL-6 was absent or low, respectively.

**Table 1:** Injection site observations in mRNA/LNP-vaccinated NHPs

mRNA dose	Pain	Erythema	Edema
1 µg (*n* = 8)	none	none	none
10 µg (*n* = 32)	none	1 × grade 1	1 × grade 1
100 µg (*n* = 16)	none	3 × grade 11 × grade 2	4 × grade 1

Injection sites were assessed for pain on palpation, erythema and edema pre-dose, at 0.5, 2, 6 and 24 h after dose and daily until day 5 after each dose or disappearance of observations. Erythemas and edemas resolved after 1 to 6 days. Scores: 0 = No; 1 = Very slight; 2 = Well-defined; 3 = Moderate; 4 = Severe. *n* = number of vaccinations

## Discussion

mRNA is a very promising and versatile vaccination approach, which has recently attracted substantial attention. We have previously demonstrated that i.d. administration of protamine-formulated prophylactic mRNA vaccines based on unmodified nucleosides can protect mice against influenza or rabies challenge infections and induces also in ferrets and pigs strong humoral responses.^7,8^ Recent studies using chemically modified mRNA revealed protective efficacy of LNP-formulated mRNA vaccines for Zika virus after i.m. administration in mice or i.d. administration in NHPs, as well as immunogenicity for influenza HA of the strains H10N8 and H7N9 after i.m. administration in NHPs and humans.^12–15^ In general, in vitro transcribed mRNA has the risk of triggering various pattern recognition receptors in the endosome (e.g., TLR7/8) or the cytoplasm (e.g., RIG-I), which lead to an anti-viral response, followed by a shutdown of translation that can lower expression of the mRNA-encoded protein.^25^ To overcome that problem, the Zika studies used mRNA harboring 1-methylpseudouridine (m1Ψ) which has been demonstrated to reduce immunostimulation and increase protein expression.^26^ However, such an unnatural modification may raise safety concerns and increase regulatory hurdles. Therefore, as an alternative approach, we used mRNAs containing only unmodified nucleosides and applied instead sequence-optimization and UTR screening to avoid inappropriate immunostimulation and to achieve high antigen expression.^16^ Nevertheless, for potency reasons it is strongly desired to equip a vaccine with a sufficient adjuvant effect, which also applies to mRNA vaccines. The specific challenge in this context is the fact that mRNA vaccines need to be taken up by the cells and require the cellular translation machinery to provide antigen, a process that can be inhibited by excessive or improper immunostimulatory activity. Therefore, it is necessary to find a combination of mRNA, supporting efficient antigen production, and formulation partner, providing sufficient adjuvant effects and enhancing cellular delivery of the nucleic acids, which does not interfere with antigen expression. Data presented in the current work demonstrate that such a combination is feasible. The specific approach we describe here yields strong immunogenicity in NHPs, which is even superior to licensed vaccines based on inactivated virus.

We observed that i.m. application of unmodified mRNA in optimized LNPs led to high expression of the encoded protein in mice, which translated into strong humoral and cellular immune responses. Several mechanisms have been described how LNPs containing ionizable amino lipids could enhance cellular delivery of nucleic acids.^27^ Firstly, they self-assemble with the polyanionic mRNA to form particles, which provide protection of the mRNA from RNases and improve uptake of the mRNA by endocytosis. Secondly, they facilitate the endosomal escape of mRNA and its access to the cytoplasm where it is translated into protein. By analyzing the injection site and the dLNs, we revealed that LNP formulation additionally led to the activation of innate immune responses, which support the induction of cellular and humoral responses. The observed increase in cellularity and the strong activation of immune cells in the dLNs even when a non-translatable mRNA was used suggest that the observed effect is independent of the encoded antigen. Interestingly, the increase in cellularity was mostly mediated by monocytes and granulocytes and may support the transport and/or presentation of the antigen in the dLNs. Further investigations are required to elucidate, whether these cells are indeed contributing to the antigen transport to the dLNs.

After demonstrating strong immunogenicity of mRNA/LNP vaccines in mice, we tested whether this also translates to NHPs, which mimic the human situation most closely. This is particularly important given the experiences with early DNA vaccines which showed promising results in small animals that were then difficult to translate into clinical success.^28^ Intramuscular vaccination of NHPs with mRNA/LNP vaccines encoding RABV-G as well as influenza HA of the strains H1N1 and H3N2 induced long lasting antibody responses, which were monitored for up to 1 year and were maintained during this period well above the titers that serve as correlate of protection. Importantly, the threshold titers were already achieved after a single vaccination suggesting that the vaccines could be used as single shot vaccines, which is especially important for vaccination campaigns in low resource settings. It should be noted that the animals were naïve regarding the vaccinated influenza strains, indicating induction of de novo immune responses after mRNA vaccination. This is in contrast to the human situation, where most adult individuals have already been primed by previous exposure to or vaccinations with heterologous strains and might respond even better to the vaccination.

An important finding of our study is that LNP-formulated mRNA can be used as a platform technology, which does not induce anti-vector immunity that can be observed with viral vectors such as adenoviruses.^29^ Instead, it allows multiple vaccinations targeting different pathogens. The animals presented in Fig. 4 were vaccinated first with the H1N1-HA mRNA/LNP vaccine and three weeks after boost with the RABV-G mRNA/LNP vaccine. Nevertheless, they mounted immune responses against both antigens. The maintenance of the H1N1-HI titers during the RABV-G mRNA vaccinations and the induction of high titers of rabies neutralizing antibodies in H1N1-HA-vaccinated animals using the same vaccine format suggest that LNP-formulated mRNA enables multiple prophylactic vaccinations. Similarly important is the observation that LNPs, which were originally developed to deliver siRNA^27^ and have already demonstrated their safety for intravenous administration in clinical trials,^30^ are also well tolerated in NHPs after i.m. administration in combination with unmodified mRNA. We observed only limited reactogenicity at the injection site and minor changes in systemic cytokine and chemokine concentrations.

mRNA vaccines can be produced relatively quickly by a generic manufacturing process, which makes them attractive in scenarios where a vaccine must be adapted to match a mutated virus sequence like the annual update of the influenza vaccine.^2^ However, they can also complement already existing vaccines which can be costly and difficult to produce. Here, we demonstrate that mRNA vaccines can compete with or even outperform licensed vaccines. The mRNA/LNP vaccines induced even CD4^+^ and CD8^+^ T cell responses against RABV-G and CD4^+^ T cell responses against H3N2-HA, which were absent in NHPs that had received the licensed vaccines Rabipur® or Fluad® and have, to our best knowledge, for prophylactic mRNA vaccines only been detected in NHPs after vaccination with self-amplifying mRNA^31^ but not with non-amplifying mRNA.

In summary, we demonstrated that intramuscular application of LNP-formulated prophylactic mRNA vaccines based on sequence-optimized, unmodified nucleosides leads to a strong induction of local innate immune responses and systemic adaptive immune responses. The vaccines were well tolerated in NHPs and induced long-lived functional antibody responses that correlated with protection for rabies and influenza virus. Notably, the humoral and cellular immune responses in NHP induced by mRNA/LNP vaccines against rabies and influenza H3N2 were superior to the licensed vaccines Rabipur® and Fluad®, respectively. These data open new avenues for accelerated vaccine development in the field of infectious diseases. In conclusion, the mRNA technology evaluated in the present study fulfills all requirements of a viable vaccine platform and thus warrants clinical testing in the near future.

## Materials and methods

### mRNA vaccines

All mRNA vaccines were based on the RNActive® platform (claimed and described in patents WO2012019780 and US20150104476). mRNA vectors contained a 5′ cap structure, 5′ UTR, open reading frame (ORF), 3′ UTR, polyA tail and did not include chemically modified nucleosides. In brief, optimization entailed GC-enrichment of the open reading frame (US20150104476) and inclusion of enhanced UTRs (WO2013143700, WO2013143698, WO2013143699). The rabies mRNA vaccine encodes the glycoprotein (RABV-G) of the Pasteur strain (GenBank accession number: AAA47218.1). Two different optimized mRNA constructs were used for immunization (RABV-G A for Figs. 3–5, and RABV-G B for Fig. 4; mRNA sequences see ref. 7) containing the same ORF but different UTRs. The influenza mRNA vaccines encode full-length HA from influenza A/Netherlands/602/2009 (H1N1) (GenBank: CY039527.2; mRNA sequence see Re6HA^8^) or A/Hong Kong/4801/2014 (H3N2) (GISAID: EPI643118; mRNA sequence available upon request). The mRNAs were produced by T7-polymerase-based in vitro run-off transcription.^32,33^ Lipid nanoparticle (LNP)-formulated mRNA was generated using LNPs provided by Acuitas Therapeutics (Canada). The LNPs used in this study are 70–100 nm particles, prepared using an ionizable amino lipid, phospholipid, cholesterol and a PEGylated lipid, and are similar in composition to the LNPs that have recently proven to be safe and efficient tools for siRNA delivery.^16,27,34^

### Animals

Mice (BALB/c, 7–9 weeks of age) were obtained from Janvier Laboratories (Le Genest-Saint-Isle, France). Experiments were approved by the Regional council Tübingen. Studies with cynomolgus monkeys (*Macaca fascicularis*) were conducted at Envigo CRS, S.A.U., Santa Perpètua de Mogoda, Spain. Animals were of Vietnamese origin, bred in captivity, nulliparous and not pregnant. Animals had at treatment start an age of 2.5–3.5 years and a body weight of 2.2–3.3 kg. All animal experiments were conducted in accordance with German (mice) and Spanish (NHPs) laws and guidelines for animal protection. Mice were vaccinated i.m. at days 0 and 21 into the tibialis anterior muscle with a single dose of 25 µl. NHPs were vaccinated i.m. at days 0 and 28 into the biceps femoris muscle with a single dose of 500 µl. Vaccination with the licensed human rabies vaccine Rabipur® (Novartis) was performed i.m. in NHPs with the full human dose according to the pre-exposure prophylaxis schedule on days 0, 7, and 28^22^ or on a reduced schedule on days 0 and 28.

### Antibody analysis

Functional anti-HA antibody titers were analyzed by HI assay. NHP sera were incubated with receptor destroying enzyme (RDEII, Denka Seiken) at 37  C overnight, inactivated (56 °C, 60 min) and incubated with kaolin. Dilutions of pre-treated sera were incubated for 45 min with 4 HAU of inactivated influenza A/California/7/2009 (H1N1) or A/Hong Kong/4801/2014 (H3N2) virus (NIBSC, UK) and 50 μl 0.5% CRBC was added. Mouse sera were treated as described.^7^ Anti-rabies VNTs in serum were analyzed by the Eurovir® Hygiene-Labor GmbH, Germany, using the FAVN test and the Standard Challenge Virus CVS-11 according to WHO protocol.

### Intracellular cytokine staining (ICS)

Induction of antigen-specific T cells was determined using ICS. PBMCs of NHPs were collected 7 days after boost vaccination, shipped over-night at 20 °C, enriched by density gradient centrifugation, and stimulated for 6 h in presence of anti-CD28 (clone CD28.2, 10 µg/ml) and anti-CD49d (clone 9F10, 10 µg/ml) antibodies and either an overlapping peptide library covering the RABV-G protein (custom made by JPT, 10 µg/peptide/ml) or media. Golgi-Plug (BD Biosciences) was added after 1 h. Cell surface staining was performed using antibodies against CD3e (APC-Cy7, clone SP34-2), CD4 (BV650, clone OKt4) and CD8 (PE-Cy7, clone RPA-T8). Intracellular staining was performed using antibodies against IL-2 (BV421, clone MQ1-17H12), IFN-γ (FITC, B27) and Granzyme B (APC, clone GB11). Aqua Dye was used to distinguish live/dead cells (Invitrogen). Cells were acquired using a Canto II flow cytometer (BD Biosciences) and flow cytometry data were analyzed using FlowJo software (Tree Star). ICS of mouse splenocytes was performed as decribed.^7^

### Cytokine and chemokine measurements

Plasma of NHPs was analyzed for inflammation biomarkers (G-CSF, IFNγ, IL-1β, IL-2, IL 4, IL-5, IL-6, IL-8 and TNF) using the Luminex-based PRCYTOMAG-40K kit (MD MILLIPORE). Serum samples and muscle or dLN protein lysates of mice were analyzed for cytokines and chemokines as described.^35^

### Flow cytometric analysis of dLNs

BALB/c mice were injected i.m. in both legs with 10 µg non-formulated or LNP-formulated F*mRNA (5′-Aminoallyl-UTP-modified mRNA labeled with Alexa Fluor® 647 NHS ester from ThermoFisher), or with buffer control. Right and left popliteal and inguinal draining LNs were isolated and analyzed by flow cytometry as described.^35^

### Statistical analyses

Analyses were performed using GraphPad Prism software, Version 6.05. Statistical differences between groups were assessed by the Mann–Whitney test.

### Data availability

The data that support the findings of this study are available from the corresponding author upon reasonable request.

## Electronic supplementary material

Supplementary Figure 1

Supplementary Figure 2

Supplementary Figure 3
