# Supplementary material for: Unmodified mRNA in LNPs constitutes a competitive technology for prophylactic vaccines
Source: NPJ Vaccines. 2017 Oct 19;2:29. doi: 10.1038/s41541-017-0032-6 (PMC5648897; doi:10.1038/s41541-017-0032-6)
Supplement: Supplementary file 1 — Supplementary Figure 1 [file 41541_2017_32_MOESM1_ESM.pdf]

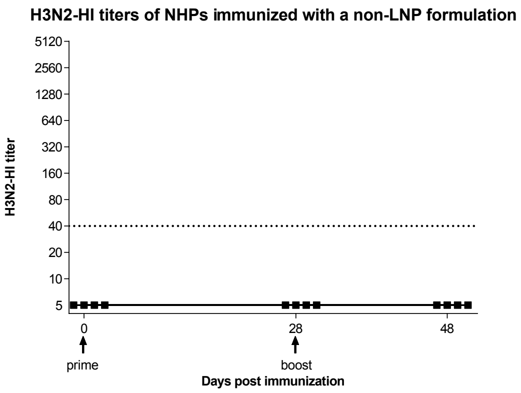

Supplementary Figure 1. Explorative H3N2-HA mRNA/non-LNP vaccine does not induce humoral responses in NHPs. The NHPs shown in Figure 4D (*n* = 2m, 2f per group), which have been initially vaccinated with 10 µg H1N1-HA mRNA/LNP, were vaccinated i.m. with 100 µg of an explorative H3N2-HA mRNA/non-LNP vaccine as indicated in Figure 4D. H3N2-HI titers were measured before and after prime vaccination with the H3N2-HA mRNA/non-LNP formulation. Values from individual animals (dots) are reported for each group. Dashed line indicates the conventionally defined protective titer of 1:40 for HI.
