# Supplementary material for: Unmodified mRNA in LNPs constitutes a competitive technology for prophylactic vaccines
Source: NPJ Vaccines. 2017 Oct 19;2:29. doi: 10.1038/s41541-017-0032-6 (PMC5648897; doi:10.1038/s41541-017-0032-6)
Supplement: Supplementary file 2 — Supplementary Figure 2 [file 41541_2017_32_MOESM2_ESM.pdf]

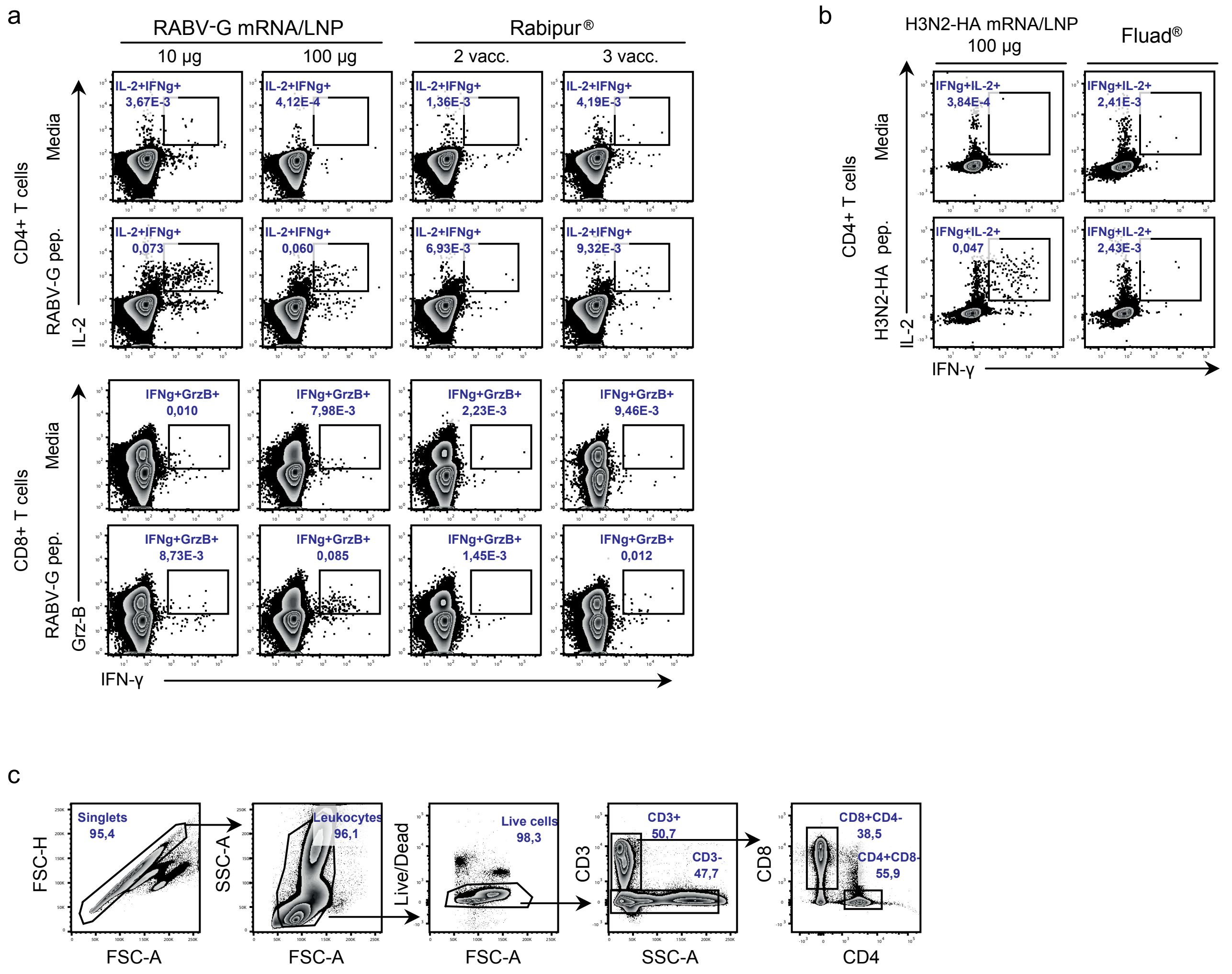

Supplementary Figure 2. Gating strategy for the detection of RABV-G- and H3N2-HA-specific T cells in vaccinated NHPs described in Figure 5. Representative dot plots are shown for each group (full data set in Fig. 5). (**a**) PBMCs of rabies-vaccinated animals were stimulated with either an overlapping peptide library covering the RABV-G protein (RABV-G pep.) or unstimulated (media), gated into $CD8^+$ and $CD4^+$ T cell populations as shown below and analyzed by intracellular cytokine staining using anti-IL-2, anti-IFN-γ, and anti-Granzyme B (Grz-B) antibodies. (**b**) PBMCs of influenza-vaccinated animals were stimulated with either an overlapping peptide library covering the H3N2-HA protein (H3N2-HA pep.) or unstimulated (media) and analyzed as in (a). (**c**) Gating of single, live, $CD3^+$ lymphocytes into $CD8^+$ and $CD4^+$ T cell populations as used in (a) and (b).
