# Supplementary material for: Unmodified mRNA in LNPs constitutes a competitive technology for prophylactic vaccines
Source: NPJ Vaccines. 2017 Oct 19;2:29. doi: 10.1038/s41541-017-0032-6 (PMC5648897; doi:10.1038/s41541-017-0032-6)
Supplement: Supplementary file 3 — Supplementary Figure 3 [file 41541_2017_32_MOESM3_ESM.pdf]

a) Cytokine concentrations in plasma of NHPs at 0h, 6h and 24h after vaccination

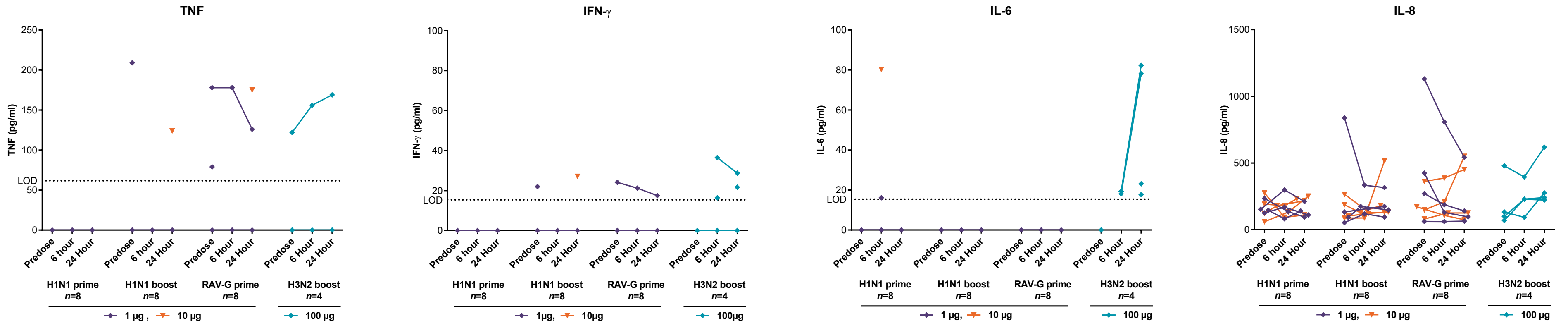

b) Body temperature of NHPs at 0h, 0.5h, 2h, 6h and 24h after vaccination

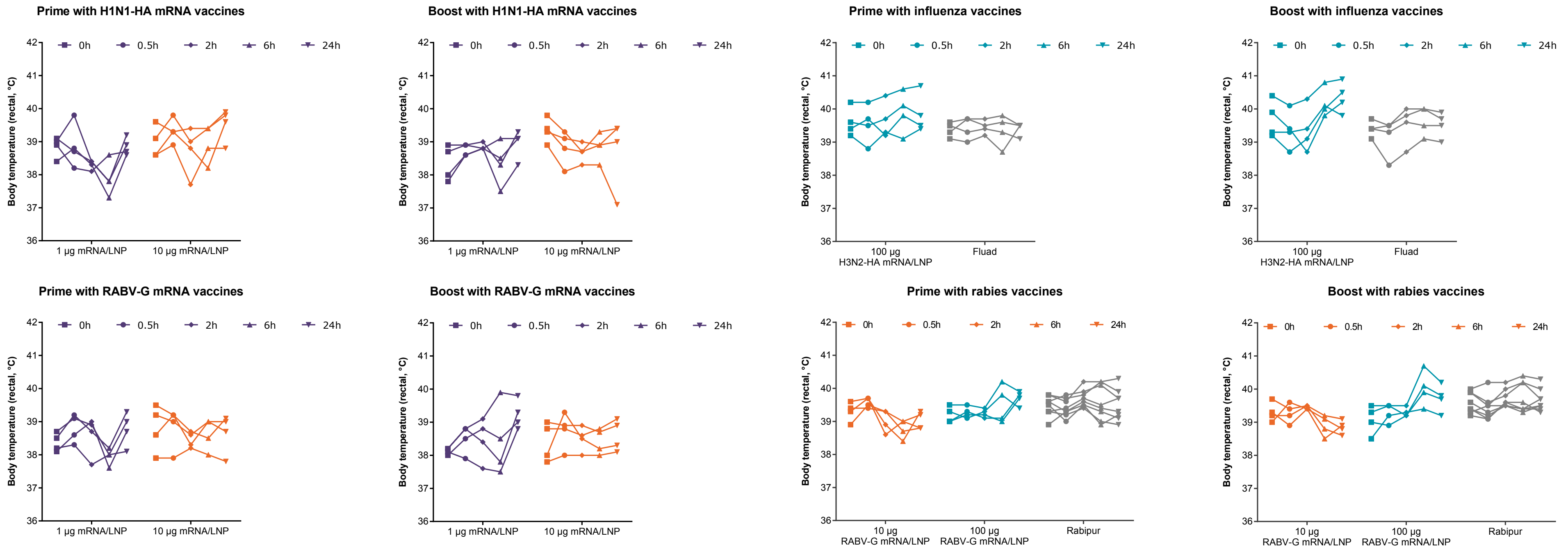

Supplementary Figure 3. LNP-formulated mRNA vaccines do not induce major changes in systemic cytokine concentrations or body temperature in NHPs. (**a**) Cytokine concentrations were measured in the plasma of NHPs of Fig. 4 before and 6h and 24h after the vaccination with 1 µg or 10 µg LNP-formulated mRNA for the H1N1-HA prime, H1N1-HA boost and RABV-G prime, as well as for other NHPs during boost vaccination with 100 µg LNP-formulated H3N2-HA mRNA. Each treatment group comprised 4 animals (2m, 2f) (**b**) Body temperature was measured in the NHPs of Fig 4 and 5 at 0, 0.5, 2, 6 and 24h after each vaccination. Values from individual animals (dots) are reported for each group. Dashed lines indicate the Level of Detection (LOD). Values below the LOD are depicted as 0.
